# SilicoDArT and SNP markers for genetic diversity and population structure analysis of *Trema orientalis*; a fodder species

**DOI:** 10.1371/journal.pone.0267464

**Published:** 2022-08-22

**Authors:** Judith Ssali Nantongo, Juventine Boaz Odoi, Hillary Agaba, Samson Gwali

**Affiliations:** National Forestry Resources Research Institute, Kifu, Mukono; National Cheng Kung University, TAIWAN

## Abstract

Establishing the genetic diversity and population structure of a species can guide the selection of appropriate conservation and sustainable utilization strategies. Next-generation sequencing (NGS) approaches are increasingly being used to generate multi-locus data for genetic structure determination. This study presents the genetic structure of a fodder species -*Trema orientalis* based on two genome-wide high-throughput diversity array technology (DArT) markers; silicoDArT and single nucleotide polymorphisms (SNPs). Genotyping of 119 individuals generated 40,650 silicoDArT and 4767 SNP markers. Both marker types had a high average scoring reproducibility (>99%). Genetic relationships explored by principal coordinates analysis (PCoA) showed that the first principal coordinate axis explained most of the variation in both the SilicoDArT (34.2%) and SNP (89.6%) marker data. The average polymorphic information content did not highly differ between silicoDArT (0.22) and SNPs (0.17) suggesting minimal differences in informativeness in the two groups of markers. The, mean observed (H_o_) and expected (H_e_) heterozygosity were low and differed between the silicoDArT and SNPs respectively, estimated at H_o_ = 0.08 and H_e_ = 0.05 for silicoDArT and H_o_ = 0.23 and H_e_ = 0.19 for SNPs. The population of *T*. *orientalis* was moderately differentiated (F_ST_  =  0.20–0.53) and formed 2 distinct clusters based on maximum likelihood and principal coordinates analysis. Analysis of molecular variance revealed that clusters contributed more to the variation (46.3–60.8%) than individuals (32.9–31.2%). Overall, the results suggest a high relatedness of the individuals sampled and a threatened genetic potential of *T*. *orientalis* in the wild. Therefore, genetic management activities such as ex-situ germplasm management are required for the sustainability of the species. Ex-situ conservation efforts should involve core collection of individuals from different populations to capture efficient diversity. This study demonstrates the importance of silicoDArT and SNP makers in population structure and genetic diversity analysis of *Trema orientalis*, useful for future genome wide studies in the species.

## Introduction

Indigenous or naturalised fodder trees and shrubs are important feed sources for livestock in a wide range of farming systems in East Africa, where over 200 000 smallholder farmers plant fodder trees [1]. In Uganda, a wide range of species are used for fodder, and have been selected based on their palatability, medicinal values and coppicing ability [2]. *Calliandra calothyrsus*, *Leucaena trichandra or Gliricidia sepium* have been the most promoted fodder species [3]. While these species have provided a basis for increased tree fodder use, promoting alternative fodder trees to supplement the current livestock feeding strategies of smallholders in mixed farming systems is key to resilience. *Trema orientalis* is a potential multi-purpose fodder. However, lack of suitable seed is still a major challenge in most fodder promotion efforts [1]. For *T*. *orientalis*, seeds are collected from the wild, where populations have dwindled, in part due to degradation of natural habitats. Herbivory may also be important in determining distribution of pioneer species such as *T*. *orientalis* [4]. This likely affects the effective sizes with consequent stochastic changes in the genetic integrity of the seeds of this promising fodder species in the wild [5,6]. Establishing the genetic structure of *T*. *orientalis* can help to establish appropriate conservation, management, and sustainable utilization strategies [7–10].

Molecular markers have become valuable tools for quantifying genetic diversity, spatial genetic structure, mating systems, gene flow and breeding patterns of tree species and many wild and cultivated plants [11]. From restriction fragment length polymorphisms (RFLPs) to simple sequence repeats (SSRs) and then to next generation sequencing of single nucleotide polymorphisms (SNPs), the types of molecular markers used to characterise genetic diversity have evolved over the past several decades [12]. However, SNPs are becoming the choice marker for genetic analysis and breeding because of the large number of markers that can be generated at a reduced cost. SNPs are also the most frequent source of variation in eukaryotic genomes and their bi-allelic nature offers accuracy in variant calling [13]. In contrast to whole genome sequencing techniques, the recent genotyping-by-sequencing (GBS) techniques such as Diversity Array Technology (DArT) (http://www.diversityarrays.com/) enables simultaneous SNP discovery and sequencing from a targeted subset of the whole-genome. The more recent DArT sequencing (DArTseq) further reduces genome representation by sequencing only the most informative representations of genomic DNA, which improves the rate of genotype calling and the ability to sequence more samples for less cost [14]. DArTseq produces dominant (SilicoDArT) and co-dominant (SNP) markers that have been successfully applied for genetic structure analysis in several crops [15,16]. The markers especially allow the characterisation of population structure without prior knowledge of the genome or diversity [17,18].

*Trema orientalis* has very few genomic resources that can contribute to its improvement and domestication. Notably, the genome has been sequenced [19], providing valuable genetic information for accurately identifying the species, clarifying taxonomy and reconstructing the intergeneric phylogeny of Cannabaceae [19]. However, knowledge of the intraspecific genetic structure of *T*. *orientalis*.is required for its management. Therefore, we used high-throughput genotyping-by-sequencing (GBS) genotyping using the DArTseq platform to assess intraspecific genome-wide diversity and population structure of *T*. *orientalis*. The objectives of this study were: 1) to assess genetic diversity in *T*. *orientalis* using SilicoDArT and SNP markers; 2) to investigate fine-scale population structure of *T*. *orientalis*. This study lays a foundation for future genome-wide association studies or genomic selection in *T*. *orientalis*.

## Materials and methods

### Study species and sample collection

*Trema orientalis* also known as *Celtis orientalis* Linn., *Celtis guineensis* Schum. and Thonn., *Trema bracteolate* Hochst Blume, *Sponia orientalis* Linn. Decne, and *Trema guineensis* (Schum. and Thonn.) Ficalho is a species of flowering tree in the hemp family, Cannabaceae [20]. It is a shrub or small to medium size tree that can grow up to 18 m high in forest regions, and up to 1.5 m tall in the savannah. The flowers are small, inconspicuous, and greenish, carried in short dense bunches. They are usually unisexual, i.e. male and female are separate, and occasionally bisexual. Flowers appear irregularly from late February to April, being pollinated by bees or wind [21]. Besides its use for fodder in Uganda as well as other African and Asian countries, the tree is useful for various wood and non-wood products [22]. *T*. *orientalis* was selected in Uganda through the National Forestry Resources Research Institute (NaFORRI), as a potential forage and therefore of interest for conservation and management.

**Fig 1:**
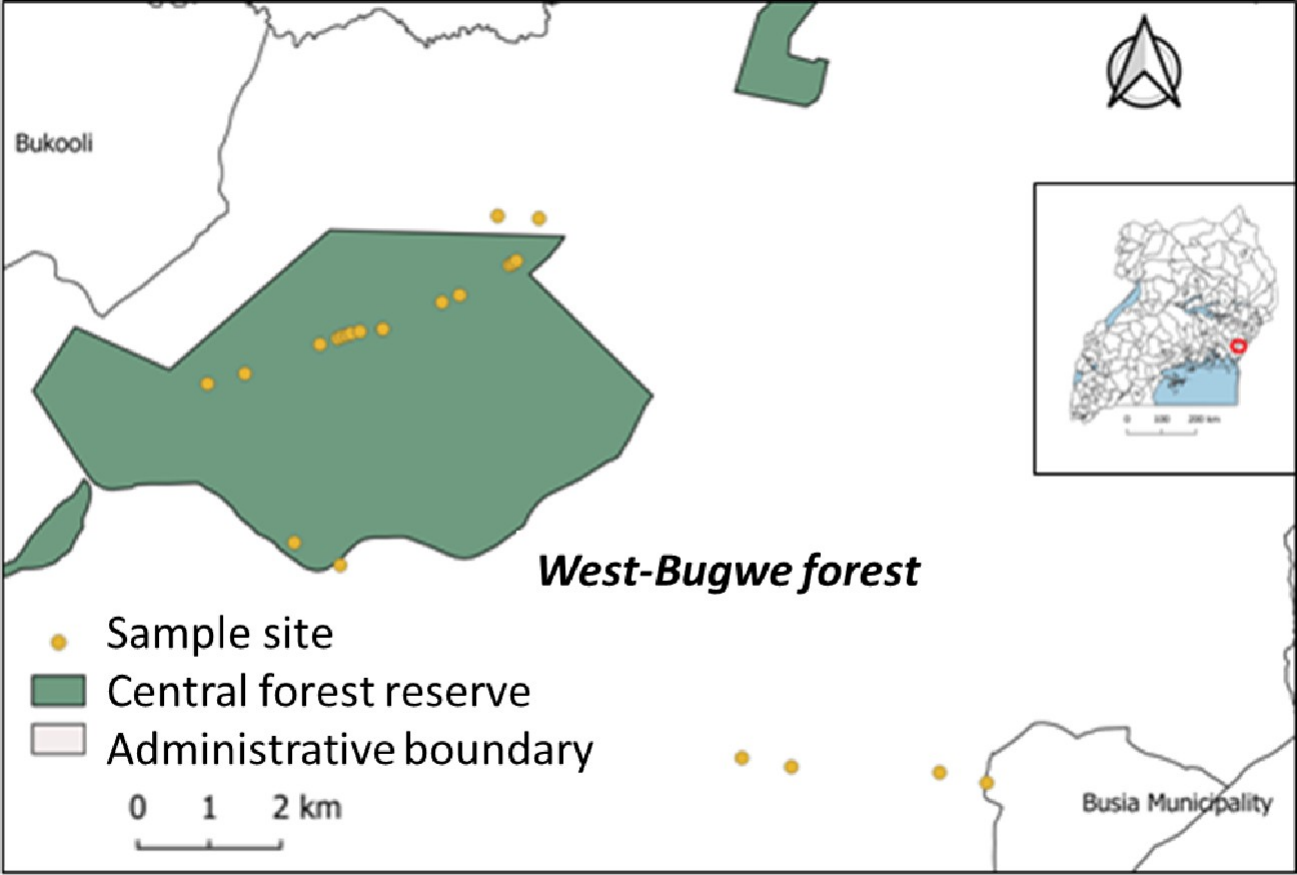
Map of Uganda (inset) and the enhanced site showing the location of West Bugwe forest and the surrounding woodlands (red mark on map) where leaf samples of *T*. *orientalis* were collected.

In Uganda, *T*. *orientalis* occurs in forest fallows especially in the Central, Eastern and Western part of the country [23]. However, most forest reserves where it occurs have been degraded, which threatens the species [24], and designing conservation strategies for priority species has been identified as a key intervention. Therefore, we characterised the genetic structure of this species, to guide in-situ conservation as well as germplasm collection for ex-situ conservation. From West Bugwe Forest reserve and the surrounding woodlands (Fig 1), 119 leaf samples were randomly collected from mature trees for DNA extraction. Upon collection, the leaves were immediately preserved with silica gel.

### DNA extraction

The leaf samples with silica gel were sent to Biosciences Eastern and Central Africa (BecA-ILRI) hub in Nairobi for DNA extraction. DNA extraction was done using Nucleomag plant genomic DNA extraction kit (Macherey-Nagel). The genomic DNA extracted was in the range of 50–100 ng/ul. DNA quality was checked on 0.8% agarose gel.

### DArTseq genotyping

DNA was shipped to Diversity Arrays Technology Pty Ltd laboratories in Canberra, Australia for processing using the DArTseq™ platform using protocol optimised for *T*. *orientalis*. DNA samples were processed in digestion/ligation reactions using a combination of PstI and HpaII Restriction Enzymes (RE) [14] with modifications, where a single PstI-compatible adaptor was replaced with two different adaptors corresponding to two different RE overhangs. The PstI-compatible adapter was designed to include Illumina flowcell attachment sequence, sequencing primer sequence and “staggered”, varying length barcode region, similar to the sequence that has been previously reported [17]. Reverse adapter contained flowcell attachment region and HpaII-compatible overhang sequence.

Only “mixed fragments” (PstI-HpaII) were effectively amplified in 30 rounds of polymerase chain reaction (PCR) using the following reaction conditions: 94°C for 1 min, 30 cycles of; 94°C for 20 sec, 58°C for 30 sec, 72°C for 45 sec, followed by a final hold of 72°C for 7 min.

After PCR, equimolar amounts of amplification products from each sample of the 96-well microtiter plate were bulked and applied to c-Bot (Illumina) bridge PCR followed by sequencing on Illumina Hiseq2500. The sequencing (single read) was run for 77 cycles. Sequences generated from each lane were processed using proprietary DArT analytical pipelines. In the primary pipeline the poor-quality sequences were filtered away. The pipeline applied more stringent selection criteria to the barcode region compared to the rest of the sequence. In that way the assignments of the sequences to specific samples carried in the “barcode split” step were very reliable. Filtering was performed on the raw sequences using the following parameters:

**Table:** 

Filter	Filter Parameters
Barcode region	Min Phred pass score 30, Min pass percentage 75
Whole read	Min Phred pass score 10, Min pass percentage 50

Approximately 2,500,000 sequences per sample were used in marker calling. Single nucleotide polymorphisms were identified by aligning reads to create clusters across all individuals sequenced. As *T*. *orientalis* is a nonmodel species, reference alleles and SNP alleles for each locus were assigned arbitrarily—in most cases, reference alleles were indicated as the allele that was most frequent across all samples for that locus. SNP markers were aligned to the reference genomes in the accessions of Chickpea_ICC_v2 and Grape_v8 of the National Centre for Biotechnology Information (NCBI) in order to identify chromosome positions. The SNPs were also aligned to several bacteria genomes to identify bacterial contamination. The BLASTN algorithm with an e-value ≤ 5e-7 and percentage identity of 90% was used. SilicoDArTs and SNPs were scored as "dominant" markers, with "1" = Presence and "0" = Absence of a restriction fragment with the marker sequence in genomic representation of the sample. SNPs were scored as codominant markers with 0 for the homozygous allele aa, 1 for the heterozygous allele Aa and 2 for the homozygous allele AA.Finally, identical sequences were collapsed into “fastqcoll files”. The fastqcoll files were “groomed” using DArT PL’s proprietary algorithm which corrects low quality base from singleton tags into a correct base using collapsed tags with multiple members as a template. The “groomed” fastqcoll files were used in the DArTs proprietary SNP and presence/absence variation (SilicoDArT) calling pipeline, DArTsoft14. For SNP calling all tags from all libraries included in the DArTsoft14 analysis are clustered using DArT PL’s C++ algorithm at the threshold sequence distance of 3 base pairs, followed by parsing of the clusters into separate SNP loci using a range of technical parameters, especially the balance of read counts for the allelic pairs. In addition, multiple samples were processed as technical replicates (from DNA to allelic calls) and scoring consistency was used as the main selection criteria for high quality/low error rate markers.

### Quality analysis of marker data

The markers were tested for reproducibility (%)–the proportion of technical replicate assay pairs for which the marker score exhibited consistency; call rate (%)–the success of reading the marker sequence across the sample; polymorphism information content (PIC)—the degree of diversity of the marker in the population and the usefulness of the marker for linkage analysis; and one ratio–the proportion of the samples for which genotype scores equalled ‘1’. The Spearman correlation between the Euclidean distances of the matrices of DArTseq and SNP markers was determined using the Mantel test in R. The raw SNP data were deposited at doi: 10.6084/m9.figshare.19181729.

### Data filtering process

The data was filtered using the dartR v 1.9.9.1 package [25] in R to remove all SNPs and silicoDArT markers that had > 5% missing data and individuals with > 10% missing data. Markers with a reproducibility score (RepAvg) < 100% were also removed as well as those that originated from the same fragment. Non-informative monomorphic markers were also removed. SNPs with a minor allele frequency (MAF) of < 1% were also discarded MAF filtration was not done for presence/absence silicoDArT. The markers were further filtered based on the one ratio value, where markers with extremely low one ratio (<0.05) were not included in the analysis.

To elaborate the genetic structure of the populations, a model-based Bayesian clustering was conducted using STRUCTURE 2.3.4 software. STRUCTURE uses a hierarchical Bayesian model to identify subpopulations and estimate global ancestry for each sampled individual based on allele frequency data [26]. The analysis was run separately for silicoDArT and SNPs. Numbers in the range from 1 to 10 were assumed for K. The initial burn-in period, for each run, was set to 100,000 with 100,000 MCMC (Markov chain Monte Carlo) iterations [27]. The admixture model was applied without using any prior population information. To find the suitable value of K, the number of clusters (K) was tested in the range from 1 to 10, and were then plotted against ΔK in STRUCTURE HARVESTER [28] to identify the most likely value of K.

Using dartR, principal coordinate analyses (PCoA) was used to investigate genetic relationships among individuals. PCoA was performed separately on the SilicoDArT and SNP datasets. To further explore the genetic relationships of *T*. *orientalis* individuals evaluated in this study, a maximum likelihood dendrogram was constructed in MEGA X using SNP markers with no prior population assumptions [29]. Using MEGA X, maximum likelihood fits of 24 different nucleotide substitution models to estimate substitution rates were developed.

### Genetic diversity analyses

Using selected markers, all genetic diversity indices were estimated using the R package “ADEGENET” [30]. The R package ADEGENET uses discriminant analysis of principal components to allow for data dimensionality reduction in large genomic datasets. The following diversity indices were therefore computed to illustrate the overall genetic divergence among the subpopulations: observed (H_o_) and expected heterozygosity (H_e_), total gene diversity (H_t_), genetic differentiation (F_st_) and population inbreeding coefficient (F_is_), fixation index (F_st_). Marker allele frequency–the frequency at which the second most common allele occurs in a given population [31], was also computed as the number of minor alleles in the population/total number of alleles in the population. Analysis of molecular variance was done using hierfstat package in R [32].

### Sequence similarity search

To put the study sequences in the context of other published sequences, 100 sequences of SNPs were randomly selected at different nodes and their similarity with published sequences searched in the NCBI database using BLASTN algorithm. A minimum e-value of 1e^-5^ and >80% identity, query coverage as well as total score were considered. Another dendrogram of *T*. *orientalis* and selected sequences from other species was generated using MEGA X [29].

## Results

### *T*. *orientalis* silicoDArT and SNP detection

A total of 4767 SNPs and 40,650 and silicoDArT markers were generated from 119 individuals of *T*. *orientalis*. The call rate of the silicoDArT markers varied between 72–100%, with an average of 98%. Missing values ranged from 5 to 10% for individual trees, and 0 to 33% for the markers. Reproducibility of the silicoDArT markers averaged to 99% (range 91% - 100%). For SNPs, missing values ranged from 0 to 50% for individual trees, and 0 to 42% for the markers. The call rate ranged from 35 to 100% with an average of 90%. The reproducibility of markers ranged from 90% to 100% with an average of 99%. The quality of marker calling was further verified by the ratio of transitions (Ts; i.e. A/G or T/C substitutions) versus transversions (Tv; i.e. A/T, A/C, T/G or C/G substitutions) which approximated to 0.5 (for both SNPs and silicoDArTs) in most of the 24 different nucleotide substitution models (S1 Table).

### Genetic diversity and Polymorphism Information Content (PIC)

**Fig 2:**
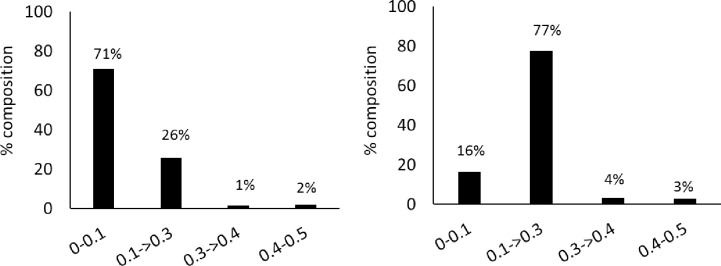
The polymorphic information content of the a) silicoDArT and b) SNP markers.

Overall, silicoDArT markers retained, the PIC value ranged from 0.02–0.5 (average = 0.22). However, there was 29% of the PIC values between 0.1–0.5 (Fig 2). The polymorphic information content (PIC) of SNPs ranged from 0 to 0.49 (average = 0.17), with 84% ranging between 0.1–0.5.

The mean minor allele frequency (MAF) based on SNPs ranged between 0.004–0.5 with an average of 0.16. Only 5% of the SNP markers had minor allele frequency less than 0.05 indicating that most markers were common genetic variants. MAF was not estimated for the dominant silicoDArT markers. After the filtration criteria above, 117 individuals were retained and 2061 SNP markers, while all individuals and 18, 163 silicoDArT markers were retained. These were used for the proceeding analyses.

The genetic diversity values calculated as expected heterozygosity (He) in the population varied from 0.05 for silicoDArTs and 0.27 for SNPs (Table 1). The low mean observed (*H*_*o*_) and expected (*H*_*e*_) heterozygosity (Table 1) corroborates with the low PIC values above.

**Table 1:** Genetic diversity of *T*. *orientalis* based on silicoDArT and SNP markers. Estimates with p indicate that these are corrected e.g. corrected Fst = Fstp.

	silicoDArT	SNPs
H_o_	0.08	0.23
H_e_	0.05	0.19
H_t_	0.06	0.40
H_tp_	0.08	0.61
D_st_	0.01	0.21
D_stp_	0.03	0.43
F_st_	0.20	0.53
F_stp_	0.33	0.70
F_is_	-0.51	-0.23
D_est_	0.03	0.52

### Population structure analysis

**Fig 3:**
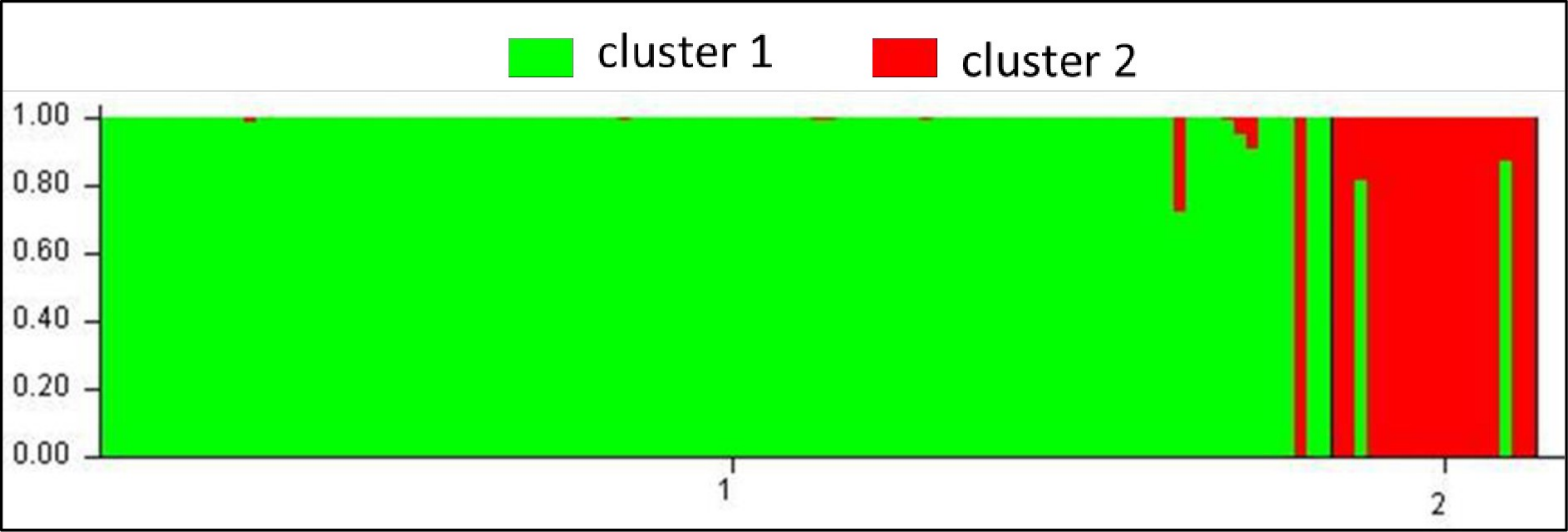
Number of clusters of the *T*. *orientalis* population using silicoDArT marker data estimated using the model-based Bayesian algorithm implemented in the STRUCTURE program. A similar graph was obtained for the SNP markers (graph not shown).

Genetic relationships among the *T*. *orientalis* individuals were assessed using a model-based clustering method that infers population structure using genotype data consisting of unlinked markers. Results from silicoDArT markers revealed 2 clusters (K  =  2) (Figs 3 and S1), where cluster I consisted of more individuals than cluster II (Table 2). Therefore, the STRUCTURE results at K = 2 were subject to population genetics analyses. Similarly, SNPs clustering revealed that there were more individuals in cluster 1 than in cluster 2. Similar clustering was also visible in the dendrogram that identified two major clusters based on SNP markers (S2 Fig).

**Table 2:** Genetic divergence among (net nucleotide distance) and within (expected heterozygosity) populations, and the proportion of membership of the population samples based on silicoDArT and SNP markers.

	silicoDArT		SNPs	
	Group 1	Group 2	Group 1	Group 2
Proportion of membership	83.7	16.3	86.2	13.8
Nucleotide distance	0.44		0.53	
Expected heterozygosity	0.09	0.20	0.06	0.26
Genetic differentiation (F_ST_)	0.76	0.55	0.96	0.55

**Fig 4:**
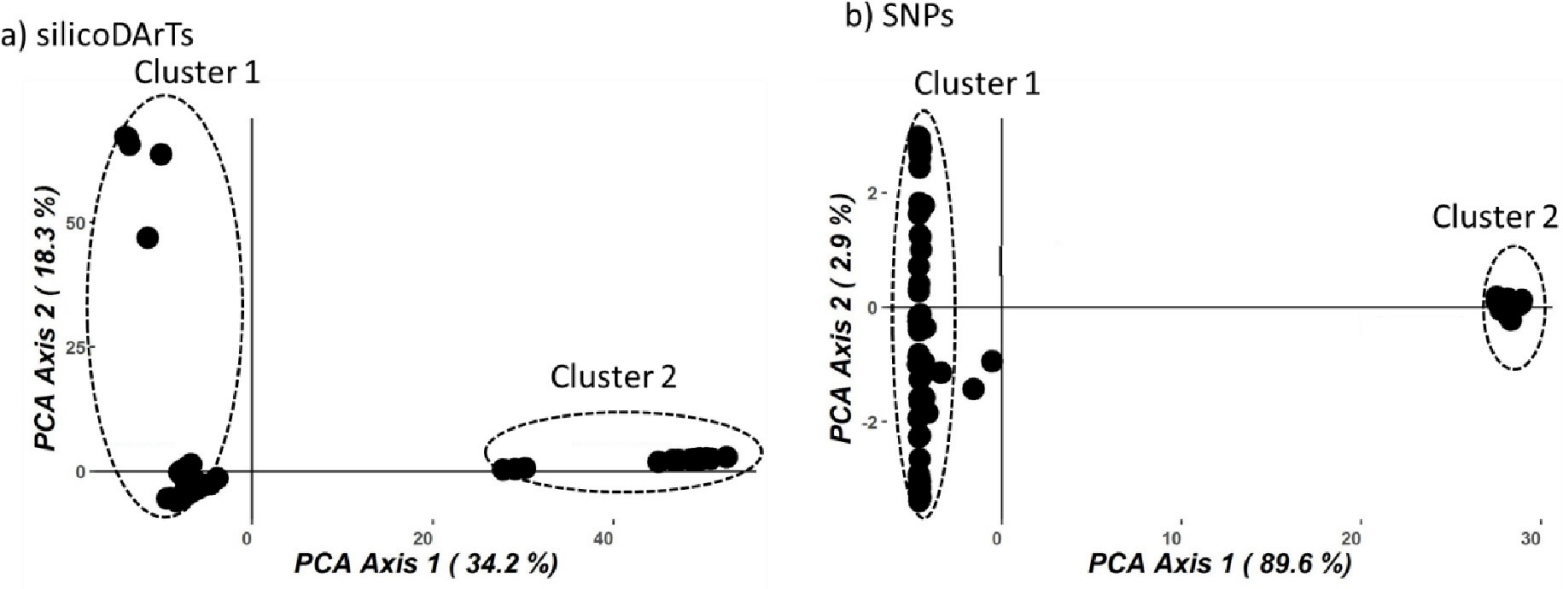
Principal coordinates analysis plot to infer group structure of *T*. *orientalis* based on a) silicoDArT b) SNP markers. Axis explained respectively 34.2% and 89.6% of the total variation in the samples based on respectively silicoDArT and SNP markers.

Genetic relationships among individuals were further explored by principal coordinates analysis (PCoA) (Fig 4). Using silicoDArT and SNP markers, PCoA identified two subpopulations, revealing the influence of tree location on the genetic diversity within *T*. *orientalis*. The first principal coordinate axis explained a higher proportion of variation (34.2% and 89.6%) than the second principal coordinate axis (18.3% and 2.9%) for both silicoDArT and SNPs (Fig 4a & 4b). For the SNP data, the clustering was tighter, and clusters had less overlap than the silicoDArT markers.

### Genetic differentiation of *T*. *orientalis*

Based on the two clusters identified in STRUCTURE, the silicoDArT markers also showed lower estimates of total genetic diversity (Ht) and genetic diversity (Dst) among groups/populations (H_t_ = 0.06, D_st_ = 0.01) compared with SNP markers (H_t_ = 0.40, D_st_ = 0.21) (Table 1). The estimates for genetic differentiation (F_st_) were also lower with silicoDArT markers (Fst = 0.20) compared to SNPs (Fst = 0.53) (Table 1). The low PIC values observed above and differences between *H*_*o*_ and *H*_*e*_ was consistent with the moderate inbreeding coefficient (F_is_), where F_is_ = -0.51[silicoDArT] and -023 [SNPs].

Overall, results indicated the presence of higher variation (AMOVA results) contained between clusters inferred using silicoDArTs (46.3%) and SNPs (60.8%) than individuals. Variation among individuals was 32.9% and 31.2% based on silicoDArTs and SNPs respectively. The consistency of these results is also reflected in the Mantel test that revealed strong association (r  =  0.61; P < 0.0001) between both markers.

### Sequence similarity

To put the resulting SNPs in the context of other sequences produced using other sequencing methods, the length of the short sequence reads corresponding with SilicoDArT markers ranged from 20 to 69 nucleotides (nt), with an average of 55.2nt and for SNPs the range was 22–69 (average 64.6 nt).

**Fig 5:**
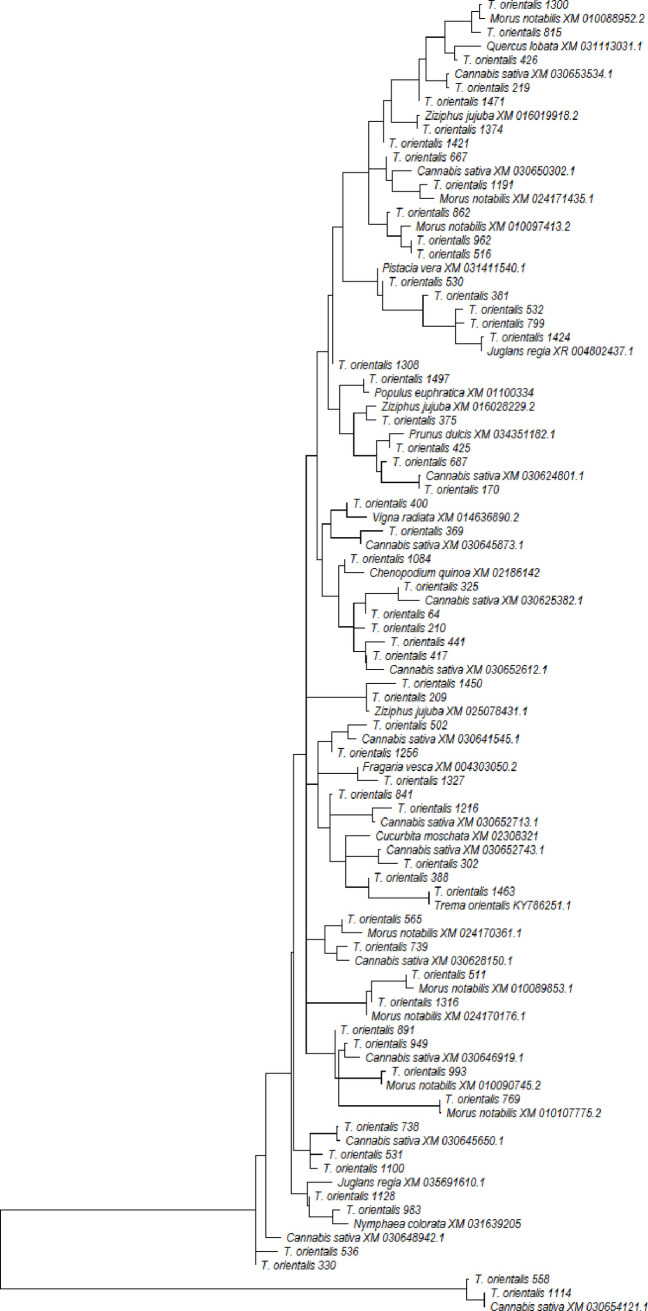
Dendrogram based on maximum likelihood showing genetic relationships *Trema orientalis* SNPs in this study and published sequences of related taxa. Sequences starting with SNP are derived from this current study while the rest are from related taxa selected from the NCBI BLAST (see methods).

Blasting the 100 sequences selected over the branches of the dendrogram, 52 SNPs could not match any other sequence, while 15 SNPs matched *Cannabis sativum* (Cannabaceae) sequences, 9 sequences matched *Morus notabilis* (Moraceae) while the rest were more similar to sequences *T*. *orientale*, *Prunus dulcis*, *Juglans regia*, *Ziziphus jujuba*, *Fragaria vesca*, *Corylus avellana*, *Vigna radiata*, *Quercus lobata*, *Populus eupratica*, *Pistacia vera*, *Chenopodium quinoa and Nymphaea colorata*. The genetic relationship among the sequences of *T*. *orientalis* and the above species is illustrated in Fig 5. The close relationship of the SNPs in this study with close members in the same lineages suggests that the identified silicoDArT and SNP markers were of high quality.

## Discussion

The importance of understanding the genetic diversity of fodder species is critical for conservation and utilization of their germplasm in breeding programs. While most studies that have used the DArT platform have mainly worked with cultivated species [16,33,34], our study highlights the suitability of DArT platform for the genomic dissection of a variety of wild plant species. Given that the average cost per data point of silicoDArT is less than SNP markers [35], the DArT platform provides opportunities for genetic-based management of diverse species in less developed countries. The DArT system enabled the detection of two types of markers, the SNPs and silicoDArT markers which; (i) exhibited high call rates and reproducibility, (ii) showed reduced genetic diversity (iii) exhibited strong genetic differentiation; and (iv) were consistent with other published sequences of taxa related to *T*. *orientalis*. Such high call rate and reproducibility has been recorded for DArT technologies in different plant species [27,36] indicating the reliability of the DArT methods for genotyping several plant species.

The results from the silicoDArT and SNP markers indicated low genetic variation in *T*. *orientalis* with potential consequences on the species ability to recover from demographic, environmental and genetic stochasticity [10]. Genetic variation in populations is measured in several ways, the most common of which has traditionally been the proportion of polymorphic loci and patterns of observed and expected heterozygosity. The polymorphism information content (PIC) values range from 0 to 0.5, where the following classification on the informativeness based on PIC values has been derived: low (0 to 0.10), medium (0.10 to 0.25), high (0.30 to 0.40) and very high (0.40 to 0.50) [37,38]. The results from the study showed that both silicoDArTs and SNPs exhibited medium to high informativeness (average PIC = 0.17–0.22) suggesting that they can detect the polymorphism among the individuals of *T*. *orientalis*. The PIC values were in the range of those established for other trees like Macadamia, where PIC for silicoDArT and SNP markers were 0.29 and 0.21 respectively, although the distribution was different [27]. The PIC values were however mostly lower than what has been detected in food crops such as beans, chickpeas, cassava and wheat [33,39–41] possibly signifying inherently low PIC values associated these markers in trees.

The average observed heterozygosity *H*_*o*_ for the markers was low but was in range of what has been reported in other tropical forest trees the same region [42,43] which could be due to anthropogenic disturbances in most natural vegetation that potentially erode the genetic diversity. However, contrary to these studies [42,43] that indicated *H*_*o <*_ *H*_*e*_, which is normally indicative of inbreeding, our study showed *H*_*o >*_ *H*_*e*_, for both SNP and silicoDArT markers. This suggests presence of an isolate-breaking effect (the mixing of two previously isolated populations or presence of hybrids) [44], consistent with the negative inbreeding coefficient that was observed for both markers, which points to presence of excessive heterozygotes. However, other hypotheses for presence of negative breeding coefficients have been highlighted [45]; including a lack of selfed progeny in small populations of outcrossing species, negative assortative mating when reproduction occurs between individuals bearing phenotypes more dissimilar than by chance and selection during the life cycle of the most heterozygous individuals. These observations are also in line with the clustering observed with both silicoDArT and SNP markers, where *T*. *orientalis* is moderately differentiated and formed 2 distinct clusters. The SNP data clustered the groups more tightly, with less overlap and explained more variation in the samples possibly because SNPs are abundant in plant genomes. This clustering was supported by results of the genetic differentiation metric (F_st_ = 0.20–0.53) between pairs of clusters. Ideally, F_st_ values below 0.05 indicate low genetic differentiation, while values between 0.05–0.15, 0.15–0.25, and above 0.25 indicate moderate, high, and very high genetic differentiation respectively [46]. The total gene diversity (H_t_ = 0.06–0.40) across markers was lower than what has been established for forest trees in the wild [43,47]. Although the mating system (unisexual flowers) of *T*. *orientalis* [22] should reduce self-fertilization, the excessive heterozygosity may be associated with restricted pollen and seed dispersal possibly resulting from fragmented landscapes [24]. The degradation may also reduce population sizes, especially the actively reproducing trees such that few trees contribute to the seedling recruitment, hence most of the trees that were sampled seemed related. Studies on the population structure and recruitment of this species in the wild are encouraged. The constraints on gene flow were also unexpected since *T*. *orientalis* disperses its seed by birds [22] and pollinated by bees which are expected to span over a large geographical area aiding the gene flow.

## Conclusion

*Trema orientalis* exhibits low genetic diversity and a potentially threatened genetic integrity. The strong population structure suggests that collection of germplasm should be done in different populations to maximise genetic variation in the collections. Characterisation of other populations is also recommended as well as studies on the population structure and recruitment of this species. The statistical analysis of DArT data sets showed high consistency with the results based on SNPs highlighting the suitability of DArT platforms for genomic dissection of *T*. *orientalis*.

## Supporting information

S1 Fig Estimation of number of groups of the *T*. *orientalis* population using silicoDArT marker data, as estimated using the model-based Bayesian algorithm implemented in the STRUCTURE program. A similar graph was obtained for the SNP markers (graph not shown).
(DOCX)

S2 Fig Dendrogram based on maximum likelihood showing genetic relationships *Trema orientalis* sequences used in this study. (DOCX)

S1 Table Maximum Likelihood fits of 24 different nucleotide substitution models. (DOCX)
