# Supplementary material for: SilicoDArT and SNP markers for genetic diversity and population structure analysis of Trema orientalis; a fodder species
Source: PLoS One. 2022 Aug 22;17(8):e0267464. doi: 10.1371/journal.pone.0267464 (PMC9394841; doi:10.1371/journal.pone.0267464)
Supplement: S1 Fig — A similar graph was obtained for the SNP markers (graph not shown). (DOCX) [file pone.0267464.s001.docx]

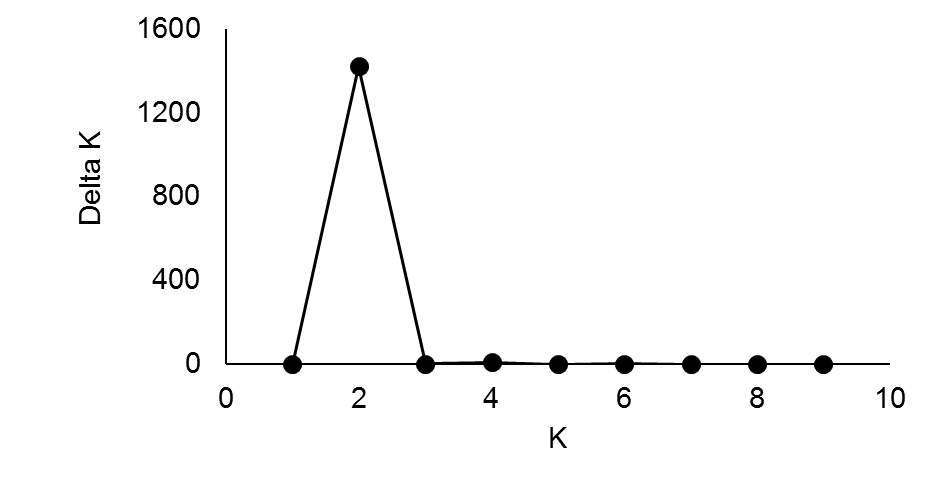


**Supplementary Figure S1.**: Estimation of number of groups of the *T. orientalis* population using silicoDArT marker data, as estimated using the model-based Bayesian algorithm implemented in the STRUCTURE program. A similar graph was obtained for the SNP markers (graph not shown).
