# Supplementary material for: SilicoDArT and SNP markers for genetic diversity and population structure analysis of Trema orientalis; a fodder species
Source: PLoS One. 2022 Aug 22;17(8):e0267464. doi: 10.1371/journal.pone.0267464 (PMC9394841; doi:10.1371/journal.pone.0267464)
Supplement: S2 Fig — (DOCX) [file pone.0267464.s002.docx]

**
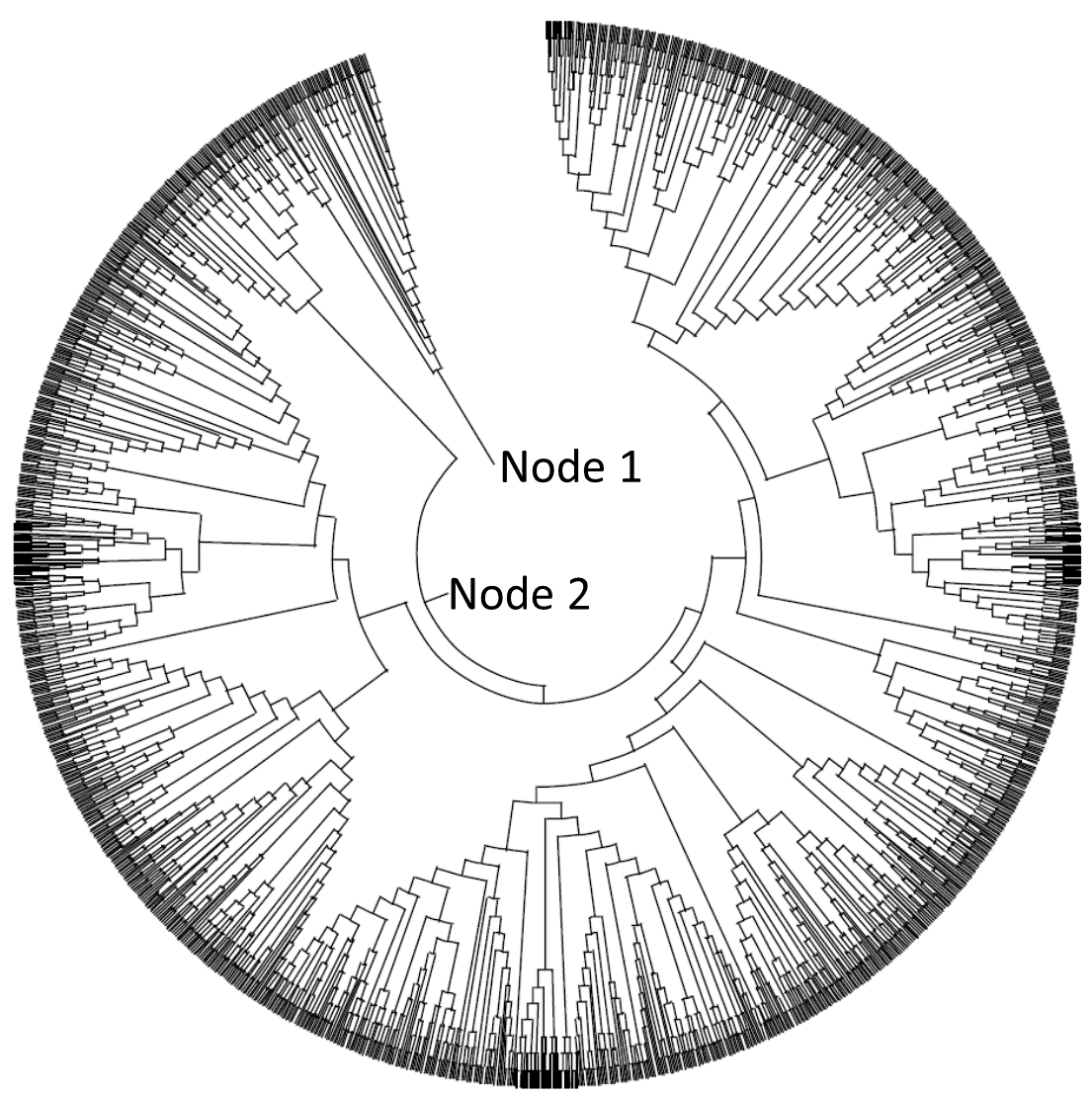
**

**Supplementary Figure S2.** Dendrogram based on maximum likelihood showing genetic relationships *Trema orientalis* sequences used in this study.
