# Supplementary material for: SilicoDArT and SNP markers for genetic diversity and population structure analysis of Trema orientalis; a fodder species
Source: PLoS One. 2022 Aug 22;17(8):e0267464. doi: 10.1371/journal.pone.0267464 (PMC9394841; doi:10.1371/journal.pone.0267464)
Supplement: S1 Table — (DOCX) [file pone.0267464.s003.docx]

**Supplementary Table, S1. Maximum Likelihood fits of 24 different nucleotide substitution models**

Models with the lowest BIC scores (Bayesian Information Criterion) are considered to describe the substitution pattern the best. For each model, AICc value (Akaike Information Criterion, corrected), Maximum Likelihood value (lnL), and the number of parameters (including branch lengths) are also presented [1]. Non-uniformity of evolutionary rates among sites may be modelled by using a discrete Gamma distribution (+G) with 5 rate categories and by assuming that a certain fraction of sites are evolutionarily invariable (+I). Whenever applicable, estimates of gamma shape parameter and/or the estimated fraction of invariant sites are shown. Assumed or estimated values of transition/transversion bias (R) are shown for each model, as well. They are followed by nucleotide frequencies (f) and rates of base substitutions (r) for each nucleotide pair. Relative values of instantaneous r should be considered when evaluating them. For simplicity, sum of r values is made equal to 1 for each model. For estimating ML values, a tree topology was automatically computed. This analysis involved 1499 nucleotide sequences. There were a total of 114 positions in the final dataset. Evolutionary analyses were conducted in MEGA X [2]

| **#Param** | **BIC** | **AICc** | **lnL** | **Invariant** | **Gamma** | **R=Ts/Tv** |
| --- | --- | --- | --- | --- | --- | --- |
| 2997 | 1546549.67 | 1518444.80 | -756125.95 | 0.00 | 0.26 | **0.50** |
| 2998 | 1546552.64 | 1518438.46 | -756121.71 | 0.00 | 0.52 | **0.48** |
| 2997 | 1546572.86 | 1518467.99 | -756137.54 | n/a | 0.50 | **0.49** |
| 2999 | 1546580.18 | 1518456.69 | -756129.76 | 0.00 | 0.25 | **0.53** |
| 3002 | 1546583.67 | 1518432.26 | -756114.34 | 0.00 | 0.47 | **0.53** |
| 2996 | 1546590.49 | 1518494.92 | -756152.08 | n/a | 0.25 | **0.50** |
| 2998 | 1546595.14 | 1518480.96 | -756142.96 | n/a | 0.50 | **0.45** |
| 3005 | 1546598.47 | 1518419.13 | -756104.57 | 0.00 | 0.52 | **0.52** |
| 3001 | 1546604.09 | 1518461.98 | -756130.27 | 0.00 | 0.57 | **0.49** |
| 3000 | 1546609.51 | 1518476.71 | -756138.70 | n/a | 0.51 | **0.46** |
| 3004 | 1546620.16 | 1518450.13 | -756121.14 | n/a | 0.50 | **0.56** |
| 3001 | 1546625.80 | 1518483.69 | -756141.12 | n/a | 0.50 | **0.49** |
| 2996 | 1569344.92 | 1541249.35 | -767529.29 | 0.00 | n/a | **0.50** |
| 2997 | 1569352.20 | 1541247.33 | -767527.21 | 0.00 | n/a | **0.51** |
| 3004 | 1569363.56 | 1541193.52 | -767492.84 | 0.00 | n/a | **0.49** |
| 2998 | 1569373.19 | 1541259.01 | -767531.98 | 0.00 | n/a | **0.50** |
| 3000 | 1569382.99 | 1541250.19 | -767525.44 | 0.00 | n/a | **0.50** |
| 3001 | 1569396.81 | 1541254.71 | -767526.63 | 0.00 | n/a | **0.73** |
| 2996 | 1569582.02 | 1541486.46 | -767647.85 | n/a | n/a | **0.50** |
| 2997 | 1569589.69 | 1541484.82 | -767645.96 | n/a | n/a | **0.50** |
| 2999 | 1569589.76 | 1541466.27 | -767634.55 | n/a | n/a | **0.50** |
| 2995 | 1569647.81 | 1541561.56 | -767686.46 | n/a | n/a | **0.50** |
| 3003 | 1569662.27 | 1541501.54 | -767647.91 | n/a | n/a | **0.47** |
| 3000 | 1569674.95 | 1541542.15 | -767671.42 | n/a | n/a | **0.78** |
